# Singing to speech conversion with generative flow

**DOI:** 10.1186/s13636-025-00400-x

**Published:** 2025-03-10

**Authors:** Jiawen Huang, Emmanouil Benetos

**Affiliations:** Centre for Digital Music, Queen Mary University of London, London, UK

**Keywords:** Singing voice conversion, Generative flow, Duration manipulation, Lyrics transcription, Phonetic similarity

## Abstract

This paper introduces singing to speech conversion (S2S), a cross-domain voice conversion task, and presents the first deep learning-based S2S system. S2S aims to transform singing into speech while retaining the phonetic information, reducing variations in pitch, rhythm, and timbre. Inspired by the Glow-TTS architecture, the proposed model is built using generative flow, with an adjusted alignment module between the latent features. We adapt the original monotonic alignment search (MAS) to the S2S scenario and utilize a duration predictor to deal with the duration differences between the two modalities. Subjective evaluations show that the proposed model outperforms signal processing baselines in naturalness and outperforms a transcribe-and-synthesize baseline in phonetic similarity to the original singing. We further demonstrate that singing-to-speech could be an effective augmentation method for low-resource lyrics transcription.

## Introduction

Research on singing analysis and manipulation using deep learning has attracted a lot of attention in recent years, including lyrics transcription and singing voice conversion. Compared to speech, singing analysis and manipulation tends to be more challenging, with pitch, duration and timbre typically varying more over time [1]. This characteristic requires greater model capacity and larger datasets for data-driven approaches.

In this work, we propose singing to speech conversion (S2S) as a novel task, which aims to reduce the variations in pitch, rhythm, and timbre in singing to create a signal that sounds like speech. The primary aim is to reduce the above variations while retaining phonetic information, rather than generating intelligible and natural speech. This distinction ensures that the converted signal retains essential phonetic information, making it potentially valuable for downstream tasks, such as being the input prompt for singing voice conversion.

As shown in [2], acoustic modeling for singing is more difficult due to these variations. Thus, S2S could potentially improve the training process for lyrics transcription by providing more uniformly structured data. In addition to its potential for analysis tasks, S2S could support singing manipulation as well. By converting singing to a normalized and interpretable format, an S2S-converted signal can serve as input for various manipulation tasks, such as singing identity conversion, pitch correction, and singing technique/style conversion. Furthermore, S2S could enhance the listening experience of individuals with hearing loss, particularly those with cochlear implants (CI). CI users perceive frequencies at a poor resolution, which may limit their enjoyment of music [3]. The pitch of a singing voice becomes more stable after S2S conversion, potentially making it easier for CI users to perceive it, leading to increased intelligibility.

Despite their natural affinity, singing and speech differ significantly in their acoustic properties. In addition to the pitch and rhythm introduced by melody, dynamics and pronunciations could be different as well [4, 5]. A previous computational pronunciation analysis study found severe confusion between the plosives “D” and “T”, and in *approximants* with vowels, when predicting phonemes in singing with a phoneme recognition model [4]. It is also common for singers to modify vowels or omit consonants for fluency in singing. These pronunciation differences affect the intelligibility of singing (i.e., words pronounced as they are in singing may be less understandable when read) but contribute to the expressiveness. We argue that the phonetic information is crucial to a singing performance, and essential to analysis and non-phonetic manipulation tasks. Therefore we focus on reducing the variations in singing attributes, while retaining the phonetic information, rather than generating high-quality, intelligible spoken lyrics.

Nowadays, automatic lyrics transcription (ALT) systems are able to achieve decent performance on monophonic singing, with a word error rate of around 10% [6] for English. Additionally, text-to-speech (TTS) systems can generate high-quality speech recordings [7]. While combining these two systems may appear to be a straightforward solution for singing-to-speech conversion, it comes with several limitations. First, this approach would result in a loss of expressiveness, as it compresses all phonetic information into text through the transcription bottleneck. Secondly, temporal information is also lost through the bottleneck, and alignment is not available between the singing and the converted signal. Thirdly, the effectiveness depends upon the quality of ALT and TTS systems, which might not be available for low-resource languages.

The contribution of this work is summarized as follows:

(I) We propose a new singing conversion task, called Singing-to-Speech, which aims at reducing variations in pitch, duration, and timbre, to create a signal that sounds like speech while retaining phonetic information.
(II) We propose the first deep learning-based S2S system, leveraging generative flow for its invertibility [8]. This enables us to perform cross-domain alignment and impose constraints on the alignment path. We tackle the challenge of drastic timing differences by adapting the monotonic alignment search algorithm to the S2S scenario.
(III) Objective and subjective evaluations show that the proposed model outperforms a signal processing-based baseline in terms of naturalness, and a transcribe-then-synthesize baseline in terms of phonetic similarity to the original singing.
(IV) We demonstrate that S2S could be an effective data augmentation method for low-resource ALT.

The rest of the paper is organized as follows: related work is presented in Section 2. The proposed S2S model is introduced in Section 3. In Section 4, we demonstrate the application of the S2S model as a data augmentation method for ALT. Experimental details are provided in Section 5. Objective and subjective results for the proposed S2S model, and the data augmentation ALT results, are discussed in Section 6. Finally, conclusions are drawn in Section 7.

## Related work

### In-domain voice conversion

Voice conversion (VC) in the context of speech or singing usually refers to changing the identity of the speaker or singer while maintaining the linguistic content. Various methods have been explored in the field of speech conversion, including using a pre-trained Automatic Speech Recognition (ASR) model to extract speaker-invariant linguistic features (text [9, 10] or phonemes [11, 12]), followed by speech synthesis for the target speaker. Others employ autoencoders to disentangle speaker and linguistic information during reconstruction using an autoencoder [13, 14]. GAN-based (generative adversarial network) models [15, 16] have been applied to alter the speaker identity while retaining the content with the help of discriminators. Singing voice conversion (SVC) research generally follows that of speech conversion: GAN-based models (ROSVC [17], FastSVC [18]) and disentanglement-based methods (VQVAE and its variants [19, 20]) have also been explored. Recent advancements have also explored the use of diffusion models and normalizing flows for SVC, demonstrating their potential to achieve high-quality conversion results [21, 22].

Other aspects beyond speaker or singer identity can also be manipulated, including pitch [23–25], singing techniques [26], and prosody [27, 28].

### Cross-domain voice conversion

It is worth noting that, although some of the above conversion pipelines involve alignment or time warping (either implicitly or explicitly), the warping does not introduce drastic changes because the conversion stays within the singing or the speech domain. In [29], the authors show that with dynamic time warping (DTW), the cross-domain (between singing and speech) alignment error is much higher than speech alignment error (between speech and speech), due to the large differences in rhythm and timing between speech and singing. It is also generally acknowledged that substantial local tempo differences are challenging for time warping methods.

As a cross-domain conversion task, S2S has a lot in common with the task in the opposite direction, i.e., speech-to-singing conversion, where speech is converted to singing given a pitch contour or a reference singing. Both tasks face challenges such as limited amounts of paired training data (singing and speech recordings with the same linguistic content) and obtaining a temporal alignment between paired singing and speech. Earlier signal processing approaches [29–31] compute the alignment between speech and singing (or/and text) using DTW or its variants. Recent studies [32, 33] have shown a growing interest in deep learning approaches without explicit synchronization input. These approaches aim to learn a mapping from speech to singing, or vice versa, without requiring explicit alignment between the two modalities.

### Flow-based generative models

A flow-based generative model [34] transforms a simple tractable probability distribution, such as a standard normal distribution, to the data distribution, through a series of invertible transformations. The resulting transformation can be applied in both directions. As a consequence, it is possible to generate new samples from the data distribution and compute the exact likelihood of existing data.

The generative process can be defined as

1 $$\begin{aligned} z \sim \pi (z) \end{aligned}$$

2 $$\begin{aligned} x = f(z) \end{aligned}$$

where *z* is a latent variable, *x* is a data sample, and $\pi$ is a tractable density, such as a multivariate Gaussian: $\pi (z) = \mathcal {N}(z;\mu ,\sigma ^{2})$. The function *f* consists of *L* invertible functions:

3 $$\begin{aligned} f=f_1 \circ f_2 \circ ...\circ f_L \end{aligned}$$

The exact log-likelihood of data *x* can be calculated as follows:

4 $$\begin{aligned} \log p(x) & = \log \pi (z) + \log \left| \det \frac{\partial f^{-1}(x)}{\partial x} \right| \nonumber \\ & = \log \pi (z) + \sum ^{L}_{i=1} \log \left| \det \varvec{\mathcal {J}}(f^{-1}_i(x)) \right| \end{aligned}$$

where $\det \varvec{\mathcal {J}}(f^{-1}_i(x))$ is the Jacobian determinant of the function $f^{-1}_i$. Flow-based models choose functions $f_i$ whose Jacobian is a triangular matrix, such that the log-determinant calculation is straightforward and efficient.

For audio generation tasks, flow-based generative models have attracted considerable interest due to their exact likelihood computation, invertibility, and efficiency [35]. Corresponding methods have been successfully used for TTS [36, 37], singing synthesis [38, 39] and voice conversion [11, 39].

### Duration prediction

Duration prediction [36, 40, 41] was proposed to address robustness issues in neural TTS models, such as word skipping, repeating and attention collapse [7]. A duration predictor either predicts the length of the whole output sequence or the length of corresponding frames in the output for each input token. While the former is used to prevent the network from generating unusually long output, the latter replaces the attention module during inference to avoid attention collapse. By removing the attention modules, misalignment caused by length mismatch between text and speech is alleviated. In Glow-TTS, duration is explicitly predicted for every input token, from which an alignment path can be generated. This exploits the inherent monotonic nature, where each input token corresponds to multiple output frames. A similar correspondence exists between speech and singing with the same linguistic content, where speech is often much shorter than singing. Given this context, we propose adopting the duration prediction from Glow-TTS, as it proves effective in handling cross-domain length mismatches.

## Proposed method

**Fig. 1:**
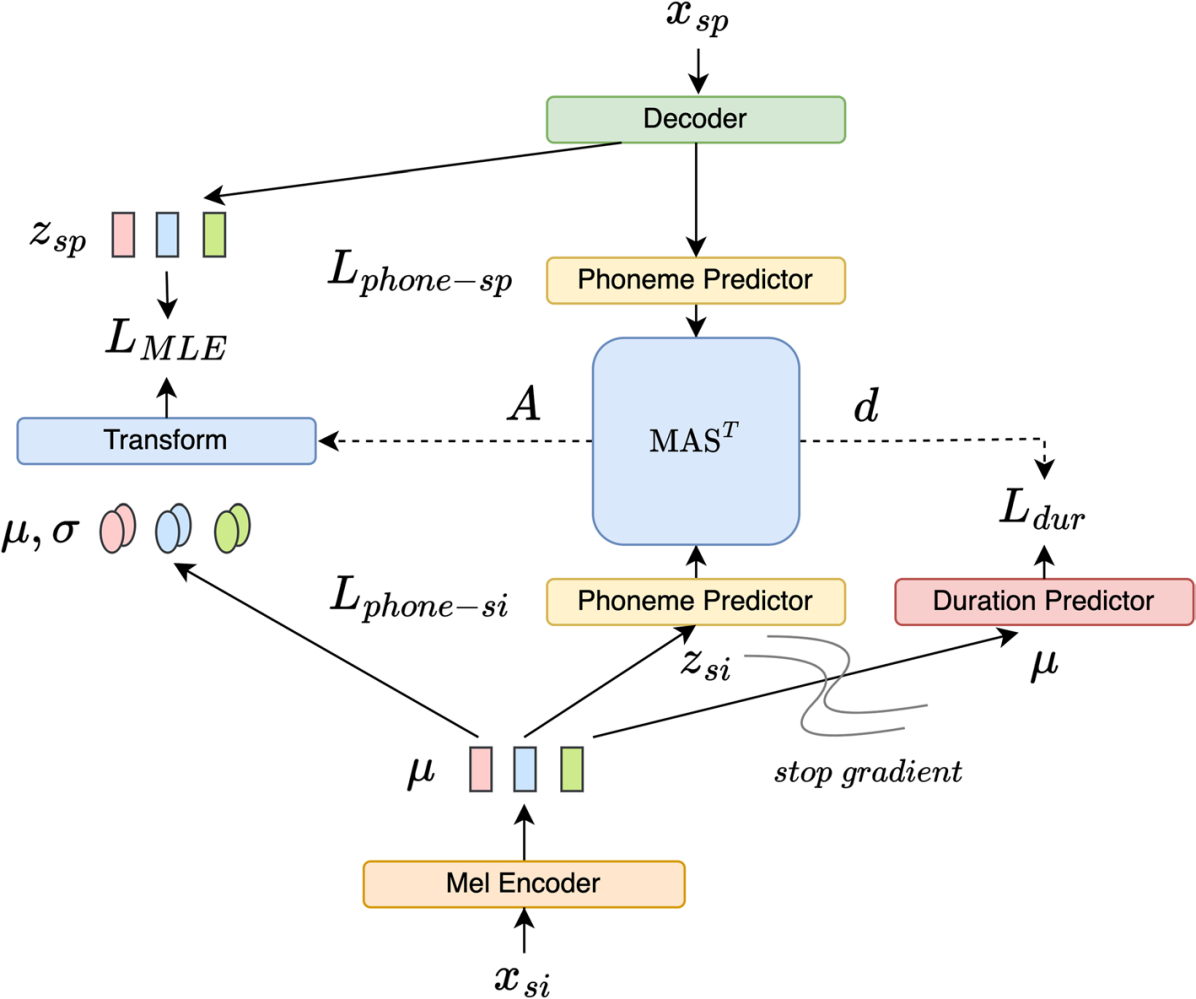
Diagram of the proposed S2S model

Our model is based on the Glow-TTS architecture [36], which in its original form aligns input text and output spectrograms using monotonic alignment search (MAS) [36] based on latent features. To adapt it to the S2S task, we need to change various core components. First, we modify the MAS algorithm to address the issue of temporal alignment between the singing and speech audio. Next, unlike TTS, S2S takes singing Mel-spectrograms as input and produces speech-like Mel-spectrograms. Thus, the proposed model consists of a Mel Encoder $f_{enc}$, a flow-based decoder $f_{dec}$, a duration predictor $f_{dur}$, and a phoneme predictor $f_{phone}$. We define $x_{si}$ and $x_{sp}$ to be a pair of singing and speech Mel-spectrograms which share the linguistic content. Let $T_{si}$ and $T_{sp}$ denote the lengths of the singing and speech Mel-spectrogram. Given the true data distribution $(x_{si}, x_{sp}) \sim p^{*}(x_{si}, x_{sp})$, the goal is to model $x_{sp} \sim p^{*}(x_{sp}|x_{si})$ (Fig. 1).

### Training

The Mel encoder $f_{enc}$ maps the singing Mel-spectrogram $x_{si}$ into the statistics of a latent space *Z*. To this end, we sample $z_{si}$ from $\mathcal {N}(\mu ,\sigma )$ with a noise level $\epsilon$ as follows. The phoneme predictor $f_{phone}$ is applied to $z_{si}$ to produce a phoneme posteriorgram $Q_{si}$:

5 $$\begin{aligned} \mu & = f_{enc}(x_{si}), \; \sigma = 1 \end{aligned}$$

6 $$\begin{aligned} z_{si} & = \mu + \epsilon \sigma \end{aligned}$$

7 $$\begin{aligned} Q_{si} & = f_{phone}(z_{si}) \end{aligned}$$

During training, an inverse pass of $f_{dec}$ maps the speech Mel-spectrogram $x_{sp}$ to the same latent space *Z*, and the same phoneme predictor is applied to produce a speech phoneme posteriorgram $Q_{sp}$:

8 $$\begin{aligned} z_{sp} & = f^{-1}_{dec}(x_{sp})\end{aligned}$$

9 $$\begin{aligned} Q_{sp} & = f_{phone}(z_{sp}) \end{aligned}$$

Let $S_{phone}$ be the phoneme sequence of the linguistic content. $f_{phone}$ is trained with a CTC Loss [42] as follows:

10 $$\begin{aligned} L_{phone-si} & = CTC(Q_{si}, S_{phone})\end{aligned}$$

11 $$\begin{aligned} L_{phone-sp} & = CTC(Q_{sp}, S_{phone}) \end{aligned}$$

According to Eq. 4, we can calculate the log-likelihood:

12 $$\begin{aligned} \log p(x_{sp}|x_{si}) = \log \pi (z|x_{si}) + \log \left| \det \frac{\partial f^{-1}(x_{sp})}{\partial x_{sp}} \right| \end{aligned}$$

Given that $z_{si}$ and $z_{sp}$ differ in length, an alignment *A* is required in some form to relate them. In Glow-TTS, the phoneme alignment is trained in the latent space *Z* with MAS. In the original MAS algorithm for TTS, it searches for the most probable monotonic alignment between the latent features computed from speech and the statistical prior distribution derived from text. Essentially, it finds a monotonic alignment path between the two, using DTW with directional constraints to ensure monotonicity. In TTS, due to the property that one input phoneme would always produce 1 or more frames, MAS does not allow multiple input tokens to be aligned with one output frame. In other words, the alignment only allows expanding the length for every token in the sequence. However, in the S2S scenario, this assumption no longer holds. Our preliminary study on the NUS-48E dataset [43] shows that around 80% of the phoneme counts are shortened in speech compared to singing. Therefore, we apply the restriction to the other direction, that we only allow shortening every input sequence. This led us to implement a variant of the MAS algorithm called MAS$^{T}$. In practice, this is implemented by simply swapping the direction restriction to ensure monotonicity in the opposite direction (shortening instead of expansion).

In [11], the authors present a voice conversion model based on Glow-TTS and mention that MAS leads to challenging training dynamics. They avoid the problem by using pre-trained explicit phoneme durations and therefore no alignment is required. In internal tests, we also found alignments in *Z*-space failing to produce reasonable alignment paths. For that reason, we compute the alignment between the singing and speech phoneme posteriorgrams $Q_{si}$ and $Q_{sp}$ using MAS$^{T}$ :

13 $$\begin{aligned} A = \text {MAS}^{T}(Q_{si}, Q_{sp}). \end{aligned}$$

Here, $A(i) = j$ encodes that the $i^{th}$ frame of $Q_{si}$ is aligned to the $j^{th}$ frame of $Q_{sp}$. From *A* we define $d_j$ as the inverse of the number of singing frames being aligned to the $j^{th}$ frame of $Q_{sp}$. This is used as the duration prediction target and the mean squared error (MSE) loss $L_{dur}$ is defined as follows:

14 $$\begin{aligned} d_j & = \frac{1}{\sum _{j=1}^{T_{si}} 1_{A(i)=j}}, j=1,...,T_{sp} \end{aligned}$$

15 $$\begin{aligned} L_{dur} & = MSE(f_{dur}(\mu ), d) \end{aligned}$$

We can describe the prior distribution as follows:

16 $$\begin{aligned} \log P(z_{sp}|x_{si}; \theta , A) = \sum ^{T_{sp}}_{j=1} log N\left( z_{sp,j}; \frac{1}{d_j}\sum _{A(i)=j}^{T_{si}}\mu _i, \sigma \right) \end{aligned}$$

where $\theta$ indicates the network parameters. The inner summation means that, when multiple singing frames are aligned with one speech frame, an average is computed among the $\mu$ values. The objective is given by:

17 $$\begin{aligned} \min _{\theta } L_{MLE}(\theta ) = \min _{\theta } -\log P(z_{sp}|x_{si}; \theta , A) \end{aligned}$$

The final loss consists of $L_{MLE}$ and $L_{dur}$, which share the same weight $\omega$, while $L_{phone-si}$ and $L_{phone-sp}$ are included without additional weighting:

18 $$\begin{aligned} L = \omega (L_{MLE} + L_{dur}) + L_{phone-si} + L_{phone-sp} \end{aligned}$$

where $\omega$ is a tunable parameter.

### Inference

During inference, we follow the same process to calculate $z_{si}$ (Eq. 5-6). Duration is predicted by the duration predictor:

19 $$\begin{aligned} d = f_{dur}(\mu +\epsilon \sigma ) \end{aligned}$$

The mapping *A* can be inferred by accumulating *d* until the sum is greater than a threshold *h*. The $z_{sp}$ is sampled from the isotropic multivariate Gaussian distribution with the aligned mean values:

20 $$\begin{aligned} z_{sp,j} \sim \pi (z_{sp,j}) & = \mathcal {N} \left( z_{sp,j}; \frac{1}{d_j}\sum _{A(i)=j}^{T_{si}}\mu _i, \sigma \right) \end{aligned}$$

21 $$\begin{aligned} x_{sp} & = f_{dec}(z_{sp}) \end{aligned}$$

### Architecture

The input $x_{si}$ is an 80-bin Mel-spectrogram, computed at 16 kHz sampling rate, with a FFT size of 1024 and a hop size of 256, from 80 to 7600 Hz. This setting is adopted to be compatible with a pretrained Parallel Wave GAN 1, which is used for speedy Mel-to-audio conversion. The Mel-spectrograms are normalized with the global mean and standard deviation.

#### Mel encoder

The first part of the Mel Encoder has 5 blocks of Conv1D layers with group normalization. The convolutional layers have 512 channels, with a kernel size of 5 and a stride of 1. Each group has 16 channels, resulting in 32 groups for normalization. The 5 Conv1D blocks are followed by another 3 Conv1D blocks, which alter the channel number from 512 to 128, 32, and 80, which is the embedding dimension and the hidden dimension for alignment. The second part uses the same architecture as Glow-TTS’s text encoder, except that the text embedding layer is removed. The output $\mu$ (and $z_{si}$) is then passed to the MAS$^{T}$ module, duration predictor, and phoneme predictor.

#### Phoneme predictor

The phoneme predictor contains 1 linear layer, 3 bidirectional LSTM layers, and 1 linear layer to map the dimension to the number of phonemes in the end. Log-softmax is applied before it is used for alignment and connectionist temporal classification (CTC) loss [42] computation.

#### Duration predictor

The duration predictor consists of 2 multi-head attention layers, accompanied by Conv1D layers and LayerNorms. Note that this is more complicated than the linear projection in Glow-TTS, because it needs to learn from the context to decide the shortening rate. Stop gradient is applied to avoid affecting other objectives.

#### **Flow-based decoder**

The flow-decoder is a stack of 12 blocks, each consisting of an activation normalization layer, invertible $1\times 1$ convolution layer, and affine coupling layer [34].

## S2S for data augmentation

Automatic lyrics trimited publicly available training data, ALT suffers from insufficient data, especially for non-English languages, as English dominates most of those datasets.

As one potential applicanscription (ALT) [44] for singing voice is the equivalent task to ASR for speech. Due to the lation, we propose S2S as a data augmentation method for ALT, which focus on low-resource settings. It has the following strengths: (1) unlike traditional augmentation methods for audio such as speed perturbation and SpecAugment [45], S2S is content-aware. Therefore, it can reduce the variations in irrelevant aspects, and let ALT focus on the phonetic information. (2) S2S does not require access to external audio and metadata. (3) S2S can be applied to unseen languages, making it suitable for low-resource language scenarios. Since languages often share numerous phonemes, an S2S model can generalize to other languages by leveraging its knowledge of phoneme mapping.

In Section 5.5, we investigate the efficacy of S2S models as data augmentation methods across varying amounts of training data and different languages.

## Experiments

### Datasets

The experiments are conducted on the DAMP Sing! 300 ✕ 30 ✕ 2 dataset [46, 47] (DSing), which contains singing recordings with lyrics annotations collected from the Smule karaoke platform across 30 countries. In each country, 300 songs are included, each performed by one female and one male singer. We use the split configuration in [47]. There are in total 81,092 (from the *DSing30* split), 482, and 480 utterances from train, development, and test splits with lyrics annotations. Because the alignment depends on the phoneme posteriorgram, it works only after the phoneme predictor is trained to a certain level. To achieve faster and more stable training, we decide to use samples that are less than 5 s and longer than 3 s. After filtering, 20,985 utterances are left for training. The lyrics are first converted to a phoneme sequence via a grapheme to phoneme tool 2.

For every singing utterance, a speech audio is generated by Tacotron2 3 [48]. This allows us to create paired training data. All of the audio files are resampled or generated at 16 kHz sampling rate.

For evaluation, we use both the test split from DSing and NHSS [49]. NHSS is a speech and singing parallel database with 2791 pairs of utterances, each has a singing and a spoken version. Although there is no direct comparison between the proposed S2S and the spoken utterances in NHSS due to our S2S task definition, the spoken utterances can serve as a source of natural prosody (intonation and rhythm) for our baselines.

### Baselines

The first baseline is the straightforward approach to S2S, which is to transcribe the lyrics and then synthesize using TTS. We choose the lyrics transcription model from [6]. The TTS model is Tacotron2 [48]. This baseline is referred to as *ALT-TTS*.

We build two more baselines with the WORLD synthesizer [50], one with duration adjustment and the other without. WORLD is a vocoder which parameterizes speech into pitch contour ($F_0$), harmonic spectral envelope (*SP*), and aperiodic spectral envelope (*AP*). It is commonly used for pitch manipulation on voices by editing the pitch contour and resynthesizing the signal [24, 25]. For baselines, we align the singing with its speech reference, and replace the $F_0$ of singing with that of the aligned reference speech. In this way, we approximate the intonation and the duration (rhythm) in the reference. We use different reference speech signals on DSing and NHSS. For DSing, the synthesized speech by *ALT-TTS* is used as a reference for intonation and duration adjustment. On NHSS, we use spoken utterances as the reference.

The steps to create the baseline with duration adjustment (*WORLD-dur*) are as follows: First, a standard DTW is applied between the MFCC features of singing and the synthesized speech, similar to the temporal alignment method adopted in [29]. Secondly, with the synchronization information, time-stretching is applied to the singing using rubberband 4. Next, the F0 contours are extracted by WORLD from the stretched singing ($F0^{align}_{si}$) and the speech ($F0_{sp}$). A linear interpolation on $F0_{sp}$ is applied to compensate the voiced/unvoiced frame mismatch due to imperfect alignment, so that the voiced/unvoiced information is the same as $F0^{align}_{si}$ for each frame. Finally, we re-synthesize the signal using the interpolated $F0_{sp}$, the stretched singing *SP*, and the stretched singing *AP*. Similar to *WORLD-dur*, if the time-stretching is applied to the synthesized speech, the output would have no duration adjustment. This is denoted as *WORLD-nodur*.

SVC models are not considered as baselines since most of them are not capable of duration manipulation, making them invalid as S2S systems for comparison.

### Training and inference

We use the ADAM optimizer, at the learning rate of 0.0001. Parameter $\omega$ is set to 10. The model is first trained for 223 epochs at a noise level $\epsilon$ of 0, and finetuned at a noise level of 0.3 for another 60 epochs. The models before and after finetuning are referred to as *M1 *and *M2*.

During inference, the duration is predicted by the duration predictor. Additionally, it is possible to manually adjust the duration by entering duration rates. When all values are set to 1, the duration adjustment is disabled, and the rhythm information in the input is preserved. To demonstrate the impact of duration adjustment on the converted audio, we compare two models: one with duration adjustment, denoted as M*-dur, and one without duration adjustment, denoted as M*-nodur.

### Evaluation

#### Objective evaluation

To evaluate the audio quality and intelligibility of the S2S systems, we adopt two objective metrics: MOSNet [51] and SRMR (speech-to-reverberation modulation energy ratio) [52, 53]. MOSNet is a deep learning-based metric that predicts a mean opinion score (MOS) estimation for voice conversion tasks. SRMR, on the other hand, is a non-intrusive speech intelligibility metric that measures the ability to comprehend speech in a noisy environment. SRMR-CI is a variant of SRMR designed for CI (cochlear implant) users, and we use it to measure the intelligibility for people with hearing loss. It is worth noting that MOSNet and SRMR may not be the most suitable metrics for evaluating the intelligibility of S2S output, because the noise distribution in our setting is heterogeneous to those from a denoising or enhancement system. Nonetheless, we believe that the combination of MOSNet and SRMR provides some useful insights into the naturalness and intelligibility. Besides, we report the word error rate (WER) and character error rate (CER) from a pretrained ASR model 5 and a pretrained ALT model [6].

#### Subjective evaluation

Our pilot subjective study shows that the model after fine-tuning (M2) generates perceptually better audio than the model without fine-tuning (M1). Due to the limited resources for a subjective test, M1 is excluded from the subjective study.

We conducted an online survey to evaluate the performance of S2S conversion methods, in which we compared five different methods: ALT-TTS, WORLD-nodur, WORLD-dur, M2-nodur, and M2-dur. We randomly selected one utterance between 3 to 5 s from each of the 8 singers (four female and four male) out of 10 in the NHSS dataset, and applied the five methods to each of these utterances. Participants in the survey were asked to rate the naturalness, phonetic similarity to the singing reference, and intelligibility to recognize the given reference lyrics on a Likert scale ranging from 1 to 5. The survey was held on Qualtrics 6.

In the context of this study, phonetic similarity refers to the extent of acoustic resemblance between two linguistic elements in terms of their pronunciations. However, it is important to note that a universally accepted definition does not exist, as pointed out in [54]. In our online survey, we define the phonetic similarity from a practical viewpoint, assessing the degree to which the given audio sample sounds like the reference in terms of pronunciation.

From our internal tests, we found that the rating of phonetic similarity can be easily influenced by audio quality and intelligibility. We observed that some participants disregarded the pronunciation and consistently chose the sample with the best quality or the most intelligible linguistic content. To reduce such biases in the analysis, we introduced two screening questions. Participants were asked to select the TTS-generated samples that demonstrated the highest phonetic similarity to a singing reference, where we modified the input phoneme prompt for TTS to mimic the singing pronunciation. Responses that did not pass the screening would be excluded from the analysis.

### S2S for data augmentation

We propose S2S to be an effective augmentation method under low-resource settings. To approximate the low-resource condition and to study how the effectiveness varies over the training data size, different training subsets from the same datasets are used.

#### Data splits

For English ALT, the three splits of DSing (DSing1, DSing3, and DSing30) are adopted. Additionally, we utilize OpenSinger [55], a Chinese singing voice dataset, to conduct parallel experiments on a language unseen during training, reflecting the model’s generalization ability. We form the test set by leaving out four singers (2 males and 2 females) out of the total 76, and another 4 singers are reserved for the validation set. We randomly sample subsets, resulting in four training sets: OS2h, OS5h, OS12h, and OS23h (named according to their total durations).

#### S2S Methods for comparison

Among the proposed S2S models, M2-nodur is selected for yielding the best ALT WER. S2S models (M2-nodur and WORLD-nodur) are compared against time warping and frequency masking from SpecAugment [45], as well as no augmentation. ALT-TTS is not included due to its dependencies on descent ALT and TTS models, which assume a high-resource setting.

#### Architecture

**Fig. 2:**
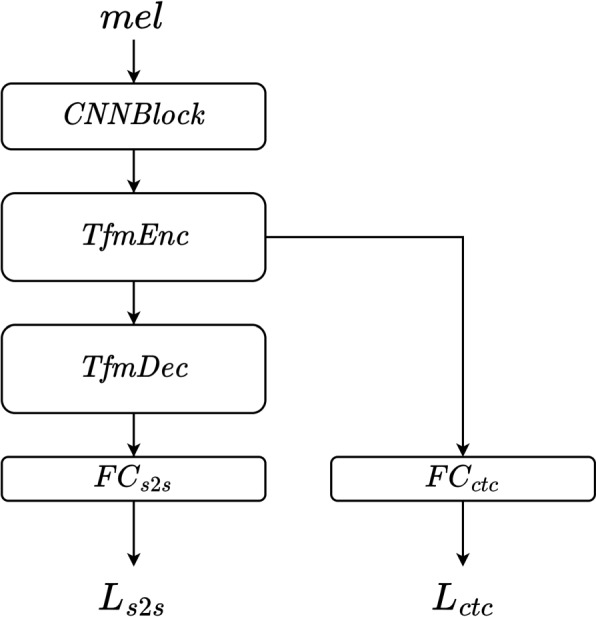
Architecture of the ALT model. It consists of a convolutional block *CNNBlock*, a transformer encoder *TfmEnc*, a transformer decoder *TfmDec*, and two fully connected layers *FC*_*ctc*_, *FC*_*s2s*_

Experiments are conducted using the same transformer ALT model and training pipeline implemented with the speechbrain [56] toolkit. The ALT model is a hybrid CTC/attention [57] transformer. Figure 2 illustrates the model architecture. The input is an 80-bin Mel-spectrogram, consistent with the proposed models. It is passed through a convolutional block and a transformer encoder. The encoder’s output is directed to two branches: one for CTC loss [42], and the other for sequence-to-sequence (seq2seq) loss. The CTC branch comprises a single fully connected layer, generating a CTC loss $L_{ctc}$. The seq2seq branch consists of one transformer decoder and another fully connected layer. The seq2seq branch produces a cross-entropy loss $L_{s2s}$ and performs inference autoregressively. The final loss is a weighted sum of the two:

$$\begin{aligned} L = \alpha L_{ctc} + (1-\alpha ) L_{s2s} \end{aligned}$$

where $\alpha$ is the weighting parameter.

We follow the default setting in a speechbrain recipe 7 and make adaptations. The input $x_{si}$ is an 80-bin Mel-spectrogram, computed at a 16-kHz sampling rate, with an FFT size of 1024 and a hop size of 256, ranging from 80 to 7600 Hz. This configuration is consistent with that of the pretrained Parallel Wave GAN model 8 and the proposed S2S model output. The convolutional block comprises 3 CNN blocks, each with 64 channels. The initial two layers have a kernel size of 5 and a stride of 2, while the last layer has both the kernel and stride set to 1. The transformer encoder consists of 12 layers, and the transformer decoder consists of 6 layers. Each encoder layer consists of a multi-head attention and a positional-wise feed-forward layer. Each decoder layer shares the same structure except that the attention layer is causal. The dimensions are configured as follows: attention dimension is set to 512, and the dimensional size of the positional-wise feed-forward layer is 2048. The number of heads is set to 8, and the loss weighting parameter $\alpha$ is set to 0.3 according to [58].

#### Training

All experiments on DSing datasets are trained for 100 epochs, and all experiments on OpenSinger datasets are trained for 300 epochs (except for OS2h is 600 epochs). The numbers are adjusted according to the convergence condition.

For both languages, we use pretrained tokenizers from huggingface hubs 9. Both tokenizers produce 5000 tokens (subwords) in total.

SpecAugment methods are applied at runtime, with the time-warping parameter set to 5, and the number of frequency masks set to 2, each with a width of 15. Augmented Mel-spectrograms using S2S methods are incorporated at training time, with each singing Mel-spectrogram having a 50% probability of being replaced by its S2S-converted counterpart.

We use the ADAM optimizer [59] and employ the Noam learning rate scheduler [60], with the initial learning rate set to 0.001. The number of warm-up steps is set to 25000. The final model is derived by averaging the best 5 validated end-of-epoch checkpoints.

For validation and testing, we apply beam search on the transformer decoder to determine the best autoregressive prediction. The beam size is set to 10 during validation and 66 during testing. No language model is involved, and we report WER for English and CER for Chinese results.

## Results and discussion

### Objective results

Table 1 shows the objective evaluation results on S2S methods and their reference audio on DSing 10. It can be observed that the MOSNet and SRMR are degraded after conversion with WORLD models, while M1 and M2 models show improvements in SRMR. This suggests that the signal processing approach introduces artifacts, while the proposed models unintentionally achieve some level of denoising and dereverberation due to the clean training target.

**Table 1:** Objective evaluation on the DSing test set

Duration adjustment	Reference		nodur			dur		
Method	Sing	ALT-TTS	WORLD	M1	M2	WORLD	M1	M2
MOSNet$\uparrow$	3.01	2.86	1.99	**2.89**	2.84	2.42	**2.96**	2.94
SRMR$\uparrow$	12.21	14.51	1.85	**15.55**	13.73	5.56	**19.86**	15.85
SRMR-CI$\uparrow$	7.40	9.08	5.08	**9.55**	8.18	4.20	**11.62**	8.93
ASR WER/CER$\downarrow$	0.54/0.31	0.31/0.18	**0.66/0.40**	1.00/0.66	0.97/0.63	0.85/0.62	0.88/0.64	**0.84/0.58**
ALT WER/CER$\downarrow$	0.13/0.07	0.22/0.15	**0.23/0.15**	0.73/0.51	0.61/0.40	0.72/0.53	0.71/0.53	**0.62/0.45**

*Sing* indicates the original singing voice

Comparing to M2, the M1 model yields superior MOSNet and SRMR results, but inferior WERs/CERs. This can be attributed to the $\epsilon = 0.3$ finetuning process, which encourages a sparser distribution of the centres of the multivariate Gaussian. This encourages the encoder to be more confident about the phoneme information, and leads to clearer pronunciation. Other $\epsilon$ values (0.1, 1.0) have been explored and we chose $\epsilon =0.3$ empirically.

All methods show an increase in WER/CER after conversion, and these results are not satisfactory. This is not surprising, partly because the ASR/ALT models are not accustomed to speech-like signals being uttered in singing pronunciations. Moreover, proposed models have limited information about semantics, hence when they encounter ambiguous phonemes in singing such as ‘D’ or ‘T’, or noisy recordings, they cannot rely on a language model, but convert it as they are. As a result, some utterances may be challenging to detect by an ASR model but still make sense to a human listener.

The ASR and ALT error rates are heavily degraded for WORLD when applying the duration adjustment, while the opposite can be observed for M1. Relatively small degradation of ALT error rates can be observed for M2. This suggests that the proposed models are more effective at addressing substantial rhythm conversion.

### Subjective results

**Fig. 3:**
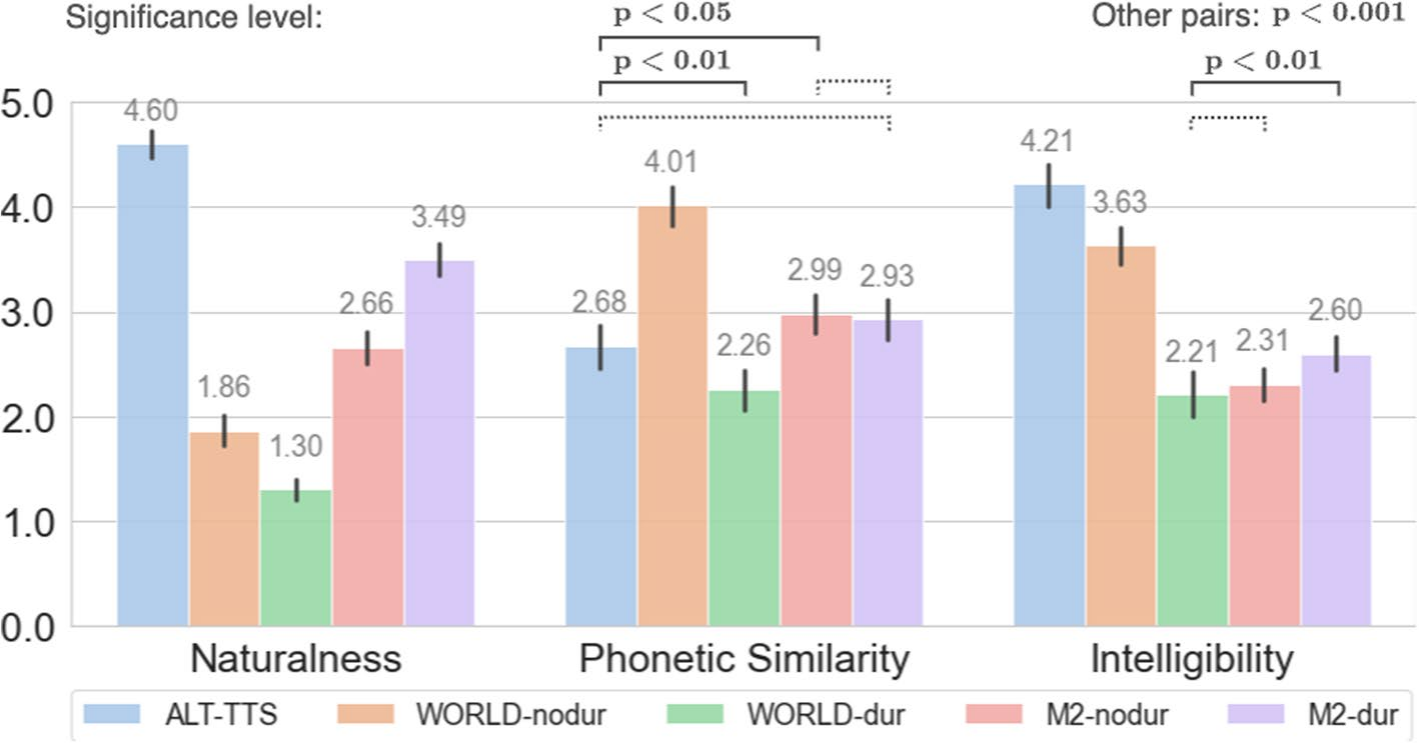
Comparison of different methods in subjective evaluation. All values are Mean Opinion Score (MOS). All pair-wise significance level is $p<0.001$ except for those indicates above the plot. A dotted line means no significance

We recruited 23 subjects who were Master’s or PhD students in Computer Science and Engineering or interdisciplinary fields involving AI. Among them, many had experience in music processing. Table 2 summarizes the participants’ self-reported gender, age, and singing experience. Figure 3 shows the MOS results of all methods and aspects, and the pair-wise Wilcoxon signed-rank test significance levels.

**Table 2:** Demographic information and singing experience of participants, including gender, age, and amount of singing experience

Gender	
Man	11
Woman	6
Age Group	
18–25	3
25–35	13
35–45	1
Singing experience	
Not trained / do not sing	6
Little training but enjoy singing	9
Trained for years	2

ALT-TTS achieves the highest score in naturalness, due to the advance of TTS technology. Our proposed M2 models outperform WORLD methods, probably because the neural network approach introduces fewer artefacts than the signal processing approach. The WORLD method performs worse with duration adjustment, while M2 performs better. This trend is consistent with the objective evaluation and is also observed in the intelligibility scores, suggesting that M2 is better at handling rhythm conversion. It is possible that alignment with time-stretching fails to produce natural and clean signals, which can negatively impact intelligibility. On the other hand, the duration predictor in M2 produces a more natural speech rhythm which is easier for human perception and contributes to a higher level of intelligibility.

WORLD-nodur achieves the highest score in phonetic similarity, probably because this is the method with the least manipulation (only in pitch) therefore the pronunciation is well-preserved. ALT-TTS is rated slightly lower than M2 models. This suggests that, despite the high accuracy of the ALT model, the ALT-TTS method may sacrifice some pronunciation details in favour of preserving linguistic content. In contrast, the M2 models retain pronunciation details better, but at the cost of a degradation in intelligibility.

### S2S for data augmentation results

**Fig. 4:**
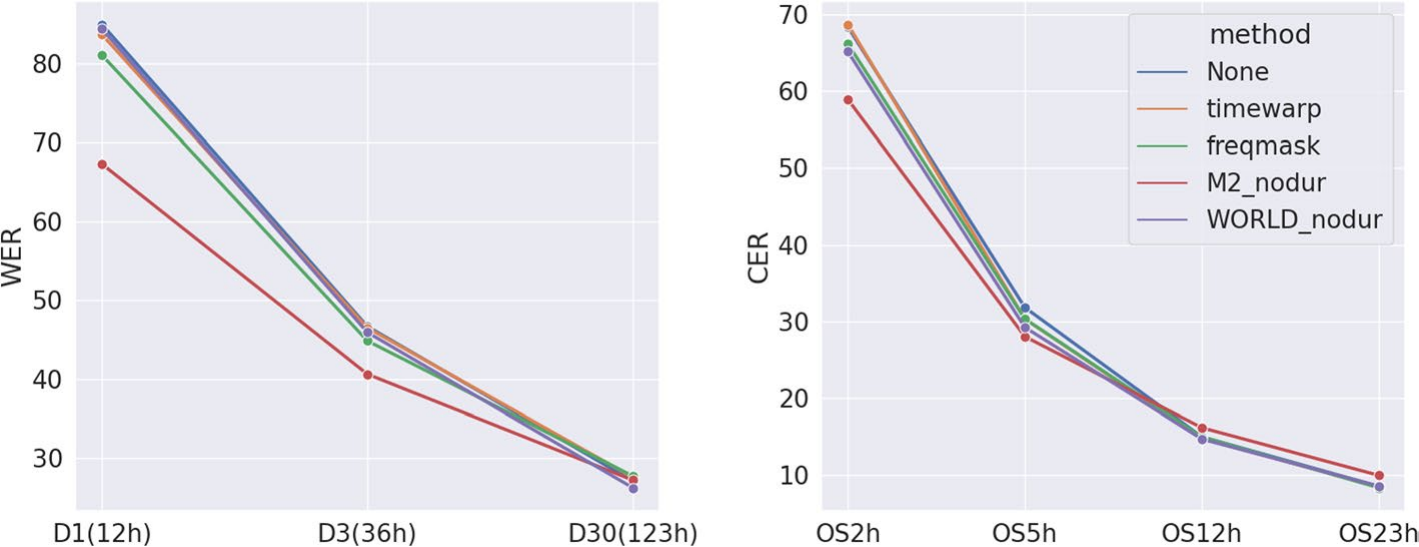
S2S for data augmentation results on different training sets. On the left: WERs on the DAMP test set, with numbers in parentheses indicating the duration of the subsets. The numbers in the parentheses indicate the duration of the subsets. On the right: CERs on the OpenSinger test set

Figure 4 displays the ALT results when training on different sizes of subsets with different augmentation methods. M2-nodur shows a clear advantage over the others under low-resource settings for both languages. As the training data increases, it performs slightly worse than the others, likely due to the error propagated from M2. It is noteworthy that M2-nodur proves effective on OS2h and OS5h, even though Chinese samples were unseen during training. Additionally, the test set involves a tonal language (Chinese), while the training data (English) is non-tonal.

## Conclusion

This work introduces the singing-to-speech conversion task and presents the first deep learning-based S2S system. By leveraging existing TTS models and technology to generate synthetic data for training and adapting the Glow-TTS model to S2S, we achieve a better balance of naturalness and phonetic similarity than the baseline models. Additionally, we demonstrate the new S2S task can benefit ALT through data augmentation, and the proposed model exhibits the ability to generalize to unseen languages. Moreover, S2S has potential as an input for various manipulation tasks and could serve to anonymize singing voices before uploading them to a voice editing service. The proposed MAS$^{T}$ may offer a solution to bridge timing differences between singing and speech. These potential advantages will be investigated in future studies.

Currently, the model is trained with $\text {MAS}^{T}$ under the assumption that all frames in singing are shortened in speech. It can sometimes lead to problematic conversion for consonants. Furthermore, glottal fricatives (“H”) are often skipped probably due to their resemblance to inhaling. To carry more accurate phonetic information, we plan to incorporate language models that leverage semantic information and make more informed decisions. On the other hand, we plan to enhance the audio quality of the converted samples by further narrowing the performance gap between M2 and ALT-TTS. This could involve refining the synthesis process to generate more life-like voice.

## Data Availability

The source code is available at https://github.com/jhuang448/singing-to-speech . The DAMP and NHSS datasets used in the experiments are accessible at https://ccrma.stanford.edu/damp/ and https://hltnus.github.io/NHSSDatabase/ with authorization. The OpenSinger dataset is available at https://github.com/Multi-Singer/Multi-Singer.github.io. The data collected from the subjective evaluation cannot be shared publicly due to ethics restrictions.

## Appendix 1: Screening questions for phonetic similarity ratings

In the context of this study, phonetic similarity refers to the extent of acoustic resemblance between two linguistic elements in terms of their pronunciations. However, it is important to note that a universally accepted definition does not exist, as pointed out in [54]. In our online survey, we define the phonetic similarity from a practical viewpoint, assessing the degree to which the given audio sample sounds like the reference in terms of pronunciation.

From our internal tests, we found that the rating of phonetic similarity can be easily influenced by audio quality and intelligibility. We observed that some participants disregarded the pronunciation and consistently chose the sample with the best quality or the most intelligible linguistic content. To reduce such biases in the analysis, we introduced two screening questions. Participants were asked to select the TTS-generated samples that demonstrated the highest phonetic similarity to a singing reference, where we modified the input phoneme prompt for TTS to mimic the singing pronunciation. Responses that did not pass the screening would be excluded from the analysis.

## Appendix 2: Objective evaluation on NHSS

In the paper, we present the objective evaluation results for the DSing test set. For completeness, results for NHSS are listed in Table 3. Since the spoken lyrics utterances are available, we use the real spoken utterances by the singer as the reference speech signal instead of TTS for WORLD baselines. Generally, Table 3 exhibits a similar trend to those on DSing test, with the exception that MOSNet and SRMRs are much higher for WORLD-nodur. This difference may be due to the use of real spoken utterances. The pitch contour extracted from the same singer’s speech provides a more natural intonation than TTS, and aligns with the normal pitch range of that speaker. For the same reason, intelligibility is better retained after duration manipulation, as reflected in WER/CERs. However, in a real-world scenario, access to the same singer’s spoken utterances is not always available.

**Table 3:** Objective evaluation on the NHSS set

Duration adjustment	Reference		nodur			dur		
Method	Sing	ALT-TTS	WORLD	M1	M2	WORLD	M1	M2
MOSNet$\uparrow$	2.96	2.88	2.56	**2.82**	2.79	2.26	2.96	**2.98**
SRMR$\uparrow$	15.20	14.76	6.86	**13.68**	12.75	4.28	**18.68**	15.59
SRMR-CI$\uparrow$	9.54	9.17	6.28	**8.74**	7.86	3.78	**10.97**	8.89
ASR WER/CER$\downarrow$	0.56/0.32	0.33/0.18	**0.65/0.39**	1.35/1.00	1.07/0.70	**0.70/0.49**	0.84/0.60	0.84/0.60
ALT WER/CER$\downarrow$	0.23/0.12	0.29/0.17	**0.31/0.18**	0.70/0.49	0.68/0.46	**0.61/0.42**	0.68/0.49	0.61/0.43

*Sing* indicates the original singing voice

## Appendix 3: Ablation study on Intelligibility

In Section 6.1 of the paper, we report the WERs and CERs with existing ASR and ALT models on the original singing, and S2S-converted singing via ALT-TTS, WORLD and proposed models **M***. We observe that all S2S methods exhibit higher WER/CER after conversion, particularly in the case of the proposed **M*** models. We argue that the degradation is partly due to ASR/ALT models not being well-acquainted with converted speech-like signals uttered in singing pronunciations. In this section, we verify this claim by quantitatively measuring the intelligibility loss caused by conversion.

Each proposed method is applied to the whole training, validation and test sets of DSing. Then an ALT model, same as described in Section 5.5.3, is trained on the converted samples. By training ALT models in their respective domains, the intelligibility metrics can accurately reflect the linguistic information extracted from the converted signals. We refer to these models as in-domain trained ALT models. The results are displayed in Table 4. The ASR and ALT results from Table 1 of the paper are also included for comparison.

**Table 4:** WER/CERs$\downarrow$ of ALT models trained in their own domains

Method	Sing	M1-nodur	M2-nodur	M1-dur	M2-dur
In-domain	0.27/0.19	0.37/0.26	0.36/0.25	0.44/0.32	0.42/0.30
ALT and ASR rows from Table 1 in the paper					
ASR	0.54/0.31	1.00/0.66	0.97/0.63	0.88/0.64	0.84/0.58
ALT	0.13/0.07	0.73/0.51	0.61/0.40	0.71/0.53	0.62/0.45

*Sing *indicates that the model is trained and tested on singing samples. *M1-nodur *means that the model is trained and tested on *M1-nodur*-converted samples. This table displays the change in linguistic information before and after conversion

In comparison to pretrained ALT and ASR models, which sometimes yield WERs close to 1, the in-domain-trained ALT models exhibit lower error rates. A WER degradation of approximately 10% is observed after conversion without duration manipulation (M*-nodur), and approximately 15% for conversion with duration manipulation (M*-dur). Interestingly, the nodur conversions outperform dur conversions for in-domain trained ALT models, contrary to the pattern observed in existing ASR and ALT models. The in-domain training results reveal that most linguistic information survived after the nodur conversion and is further degraded through duration manipulation. The poor existing ASR/ALT results can be attributed to the domain mismatch between their training and the S2S target.

## Footnotes

1 https://github.com/kan-bayashi/ParallelWaveGAN

2 https://github.com/Kyubyong/g2p

3 https://github.com/coqui-ai/TTS

4 https://breakfastquay.com/rubberband/

5 https://huggingface.co/speechbrain/asr-transformer-transformerlm-librispeech

6 https://www.qualtrics.com/

7 https://github.com/speechbrain/speechbrain/blob/v1.0.0/recipes/LibriSpeech/ASR/transformer/hparams/transformer.yaml

8 https://github.com/kan-bayashi/ParallelWaveGAN

9 speechbrain/asr-transformer-transformerlm-librispeech for English and speechbrain/asr-transformer-aishell for Chinese.

10 The results on NHSS are presented in appendix 2 and exhibit a similar trend.
